# Nutrient Status of Vitamin D among Chinese Children

**DOI:** 10.3390/nu9040319

**Published:** 2017-03-23

**Authors:** Shuojia Wang, Guosong Shen, Shuying Jiang, Hongwei Xu, Minchao Li, Zhaopin Wang, Su Zhang, Yunxian Yu

**Affiliations:** 1 Department of Epidemiology & Health Statistics, School of Public Health, School of Medicine, Zhejiang University, 866 Yu-Hang-Tang Road, Xihu District, Hang Zhou 310058, Zhejiang, China; Wangsj2015@163.com (S.W.); jiangshuying91@163.com (S.J.); bookfandingyuehao@163.com (H.X.); liminchao@zju.edu.cn (M.L.); zpwang7891@126.com (Z.W.); 2 Prenatal Diagnostic Center, Huzhou Maternal & Child Care Hospital, Huzhou 313000, Zhejiang, China; hzfbysgs@163.com (G.S.); hzfbyzs@126.com (S.Z.)

**Keywords:** vitamin D, deficiency, insufficiency, children, adolescent

## Abstract

Background: Vitamin D deficiency is considered to be a public health problem. However, the nutrient status of vitamin D in Chinese children is unclear. The aim of this study was to describe the vitamin D status among children aged under 18 years in southeast China. Methods: Children who visited the Huzhou Maternal and Child Care Hospital from January 2012 to August 2015 were included in this large cross-sectional study. Serum 25-hydroxyvitamin D (25(OH)D) concentrations were measured by electrochemiluminescence immunoassay. Vitamin D status was defined as deficiency (25(OH)D < 20 ng/mL), insufficiency (25(OH)D: 20–29 ng/mL) and sufficiency (25(OH)D ≥ 30 ng/mL). The association between relevant variables and vitamin D status was analyzed by a using generalized estimated equation model and a multivariate regression model. Results: 13,997 children aged under 18 years were included. Of these, 23.3% children suffered from low vitamin D status (deficiency and insufficiency), while 76.7% had a sufficient vitamin D status. The prevalence of low vitamin D status was 29.7% in winter and 23.4% in spring, which was higher than that in summer (21.4%) and autumn (19.9%). Clinical visiting children (32.1%) suffered more from low vitamin D than health examination children (17.6%). Additionally, age and season were independently and significantly associated with 25(OH)D concentrations, respectively. Conclusions: The deficiency and insufficiency status of vitamin D was very common among newborns and children aged one to 17 years. This indicates that more sunshine and vitamin D–fortified foods are necessary among Chinese children.

## 1. Introduction

There is growing concern about vitamin D status (serum 25-hydroxyvitamin D (25(OH)D) concentration). Vitamin D deficiency is becoming a public health problem in both developed and developing countries [1,2,3]. It is estimated that more than one billion people have suffered from insufficient vitamin D [4]. Vitamin D deficiency is related to numerous adverse health issues, besides skeletal effects, and various chronic diseases, such as common infections, cancer, autoimmune and cardiovascular diseases, are also related to serum 25(OH)D concentrations. It has been reported that vitamin D deficiency in infancy is suspected to lead to type 1 diabetes in adults [5], and there is a strong association between nutritional rickets and pneumonia [6]. Simultaneously, the prevalence of vitamin D deficiency is associated with metabolic syndromes in children [7]. Therefore, greater attention has been paid to vitamin D status during childhood.

The high prevalence of vitamin D deficiency was reported in northern or southern regions, among adults and children. A recent study in Nepal found over 90% children aged one to five years have vitamin D deficiency (<20 ng/mL) between September and November [8]. A study in the UK suggested that up to 70% of adolescents aged 14.7 to 16.6 years were vitamin D deficient (<12 ng/mL) in May [9]. In the Netherlands, 6.2% of the children aged six years were severely vitamin D deficient (<10 ng/mL), 23.6% were deficient (10–20 ng/mL), and among these children, the prevalence of vitamin D deficiency was higher in African, Asian, Turkish and Moroccan children than that in those of a western ethnic background. The prevalence of vitamin D deficiency was higher in winter (51.3%) than in summer (10.3%) [10]. Nevertheless, vitamin D deficiency is not limited to countries with less sunshine; in countries with plenty of sunshine, such as Greece, 47% of children aged 15–18 years and 14% aged 3–14 years also suffered from vitamin D deficiency (<10 ng/mL) [11]. In China, a large-scale cross-sectional survey in Beijing (40° N Latitude) showed that 45.2% of adolescent girls had plasma 25-hydroxycholecalciferol values less than 5 ng/mL in winter and 6.7% in summer [12]. Another study in Shanghai (31° N latitude) showed that almost one-third newborns had 25-hydroxyvitamin D (25(OH)D) less than 20 ng/mL [13]. Serum 25(OH)D < 20 ng/mL and 25(OH)D < 10 ng/mL occurred in 10.5% and in 1.3% of healthy children under 10 years of age in Nanjing (32° N) from December to March [14]. A study in southeast China found that vitamin D deficiency (<20 ng/mL) and insufficiency (20–29 ng/mL) were prevalent among infants, preschool children, school children and adolescents in Wenzhou [15].

Previous studies have evaluated vitamin D status in young children and adolescents, but most of them focused only on several age groups of children with small sample sizes [13,14,16]. Children's vitamin D status in this region of China has not been studied at the population level. Therefore, the purpose of this study with a large sample size was to describe the vitamin D status among children aged from one day to 17 years in southeast China.

## 2. Participants and Methods

### 2.1. Study Population

Huzhou is situated in southeastern China at latitude of 30.86° N and characterized by a subtropical monsoon climate. It has abundant precipitation and plenty of sunshine (1871.73 h/year). According to the Sixth National Population Census, the resident population in Huzhou was 2.89 million; where 11.67% people aged 0 to 14 years old and 72.12% people aged 15 to 59 years old. The economic level of Huzhou is higher than the national average. 

The study was a large hospital based cross-sectional study. Children aged less than 18 years visited in Huzhou Maternal and Child Care Hospital from January 2012 to August 2015 were included in this study. From the decoded dataset of the hospital, we extracted the information including age, gender, visiting date, visiting type (defined as ‘health examination’ or ‘clinical visiting’), and concentration of 25(OH)D. The children who went to hospital for health examination or seeking clinical service were defined as health examination and clinical visiting, respectively. The study protocol was approved by the Medical Ethical Committee of Zhejiang University School of Medicine.

### 2.2. 25-Hydroxyvitamin D Measurement

The 25-hydroxyvitamin D measurement is a routine examination in children’s health examination. First, 2 mL venous blood samples were collected after 8 h fasting. The blood samples were then centrifuged at 3600× *g* for 3 min. The serum was separated and stored at −80 °C before analysis. Serum 25(OH)D concentrations were measured with Roche Elecsys modular analytics Cobas using an electrochemiluminescence immunoassay (Roche Diagnostics GmbH, Mannheim, Germany). Precision testing showed within-run coefficient of variations (CVs) of ≤7%, within-laboratory CVs of <9.5%, between-laboratory precision CVs of ≤10.1% [17]. Quality Control materials were analyzed every day to test if the measuring value was within analytical measuring range. Standard control was used for adjustment of measuring curve when the batch of reagent was changed.

### 2.3. Assessment of Vitamin D Status

Circulating 25(OH)D concentration was used for evaluating an individual’s vitamin D status [18]. Vitamin D status, defined as deficiency (25(OH)D < 20 ng/mL), insufficiency (25(OH)D: 20–29 ng/mL) and sufficiency (25(OH)D ≥ 30 ng/mL) respectively, according to the Endocrine Society clinical practice guidelines [19].

### 2.4. Statistical Analyses

The age variable was categorized into seven groups (zero to three months, four to six months, seven to 12 months, one to three years, four to six years, seven to 10 years, 11–17 years).

The sampling season was defined as: spring (March to May), summer (June to August), autumn (September to November) and winter (December to February). 

Mean and standard deviations (mean ± SD) were used to express continuous variables. Frequencies and percentages (%) were reported for the categorical variables. Chi Square test was used to compare the difference in prevalence of vitamin D status (categorical variable) in various groups. Continuous variables were compared using analysis of variance (ANOVA). Meanwhile, generalized estimated equation modeling was used to detect the association of gender, season, age and visiting type with serum 25(OH)D concentrations considering repeated measurements. Multinomial logistic regression analysis was performed for those variables with vitamin D status (sufficient (>30 ng/mL), insufficient (20–30 ng/mL) and deficient (<20 ng/mL)). In order to compare the difference in vitamin D status between children of health examination and clinical visiting, and also to compare their results with the overall population, we repeated multiple regression models after stratifying by visiting type. Considering the effects of gender, season, age and visiting type on vitamin D status, all four variables included in the same models. Additionally, the figures were generated using the 25(OH)D concentration/percentage of low vitamin D respectively, for age groups stratified by gender, visiting type and season. All statistical analyses were performed by using the program package SAS version 9.4 (Institute, Inc., Cary, NC, USA). The *p* less than 0.05 was considered as statistically significant difference.

## 3. Results

A total of 13,997 children aged one day to 17 years were included in this study, including 7739 boys and 6258 girls. Of them, 8499 went to the hospital for a health examination, while 5498 for were there for a clinical visit. Overall, 23.28% children suffered from low vitamin D status (<30 ng/mL), while 6.43% had vitamin D deficiency (<20 ng/mL). When children were greater than one year old, the prevalence of vitamin D insufficiency and deficiency increased sharply with age.

### 3.1. Age and Serum Vitamin D Status

**Figure 1:**
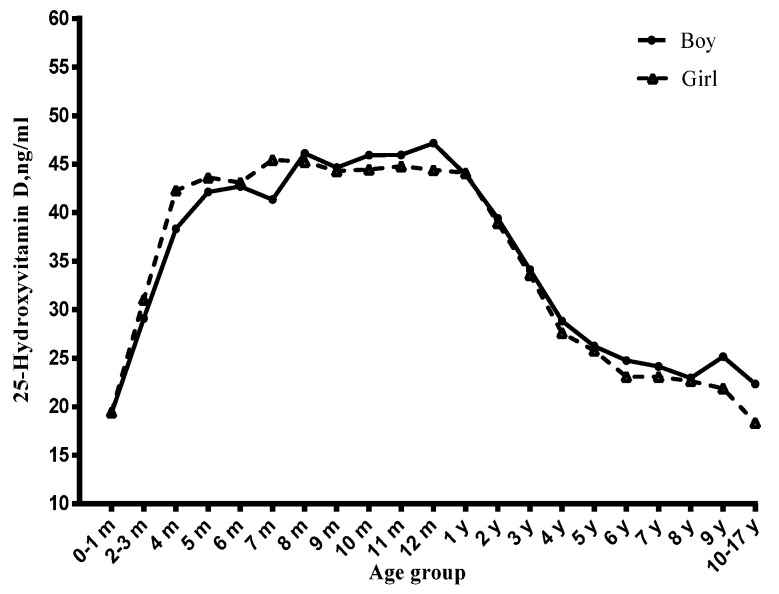
Distribution of serum 25-hydroxyvitamin D concentration by age, stratified by gender.

**Figure 2:**
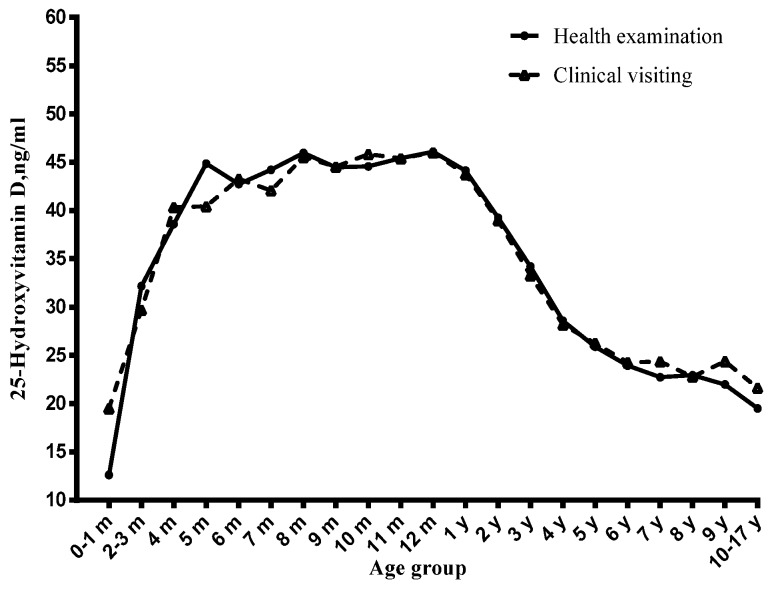
Distribution of serum 25-hydroxyvitamin D concentration by age, stratified by visiting type.

**Figure 3:**
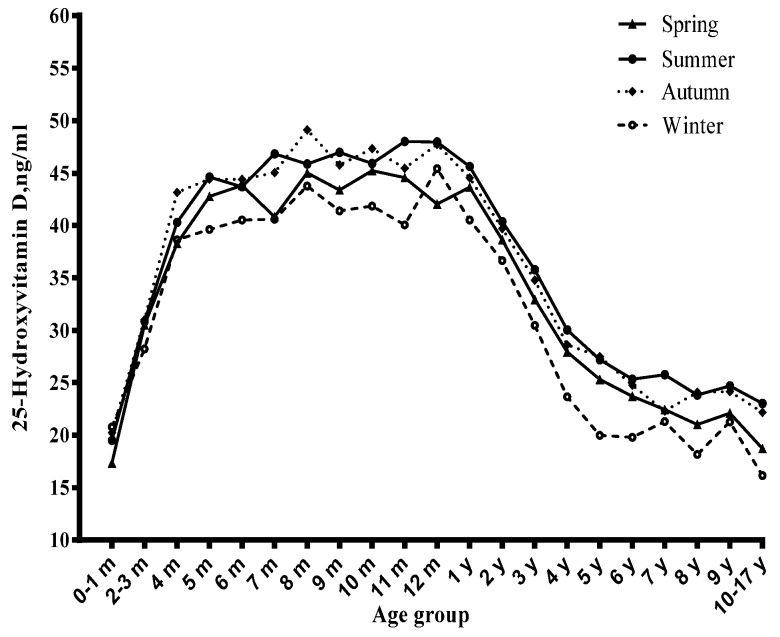
Distribution of serum 25-hydroxyvitamin D concentration by age, stratified by season.

Among children, the vitamin D status significantly varied with age (Figure 1, Figure 2 and Figure 3). Before six months, serum 25(OH)D concentrations significantly increased with the age month, and reached a peak at six months. From six to 12 months, the serum 25(OH)D concentrations stayed at the peak level. However, from one year to 17 years, it decreased continuously and significantly with age. Compared with newborn children, children aged four to six years, seven to 10 years and 11~17 years had lower serum 25(OH)D concentrations and had a higher risk of vitamin D deficiency (Table 1), especially in the 11–17 years group (OR = 14.61, 95% CI: 6.41–33.27) (Table 2). 

### 3.2. Season and Serum Vitamin D Status

**Figure 4:**
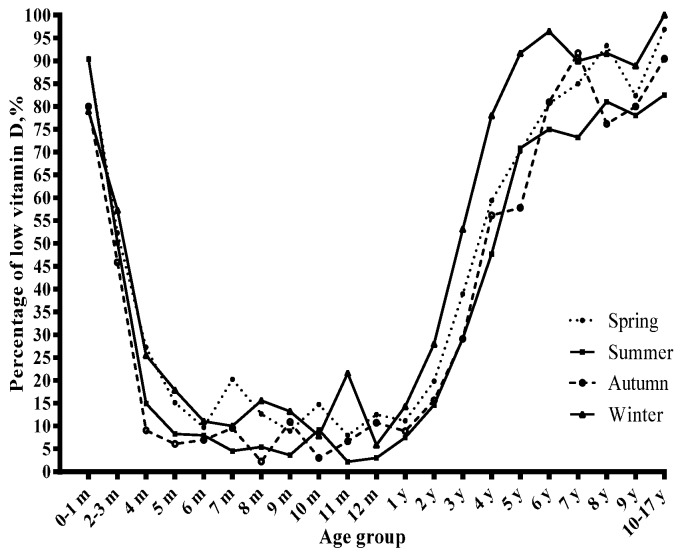
The percentage of low vitamin D (vitamin D deficiency and insufficiency) stratified by season.

The concentrations of serum 25(OH)D varied with season (Figure 3), and the prevalence of vitamin D insufficiency and deficiency showed the opposite trend (Figure 4). The prevalence of vitamin D deficiency was higher in winter and spring (9.3 and 7.1%, respectively) than in summer and autumn (5.2% and 4.7%, respectively) (Table 3). Winter was related to lower serum 25(OH)D concentrations and a higher risk of vitamin D deficiency than the corresponding reference group (Table 1 and Table 2).

### 3.3. Visiting Type and Serum Vitamin D Status

Vitamin D insufficiency occurred in 13.8% of the health examination children and in 21.6% of the clinical visiting children, and deficiency occurred in 3.8% of the health examination children compared to 10.5% of the clinical visiting children (Table 3). The health examination children had higher serum 25(OH)D concentrations than the clinical visiting children (41 ± 11 ng/mL vs. 37 ± 13 ng/mL) (Table 1). The risk of vitamin D deficiency and insufficiency was higher in clinical visiting children than the corresponding reference group. The results in the health examination children and clinical visiting children were similar to those in the whole sample (Tables S1–S3). 

### 3.4. Gender and Serum Vitamin D Status

The mean serum 25(OH)D concentrations were 40 ± 12 ng/mL in girls while they were 39 ± 12 ng/mL in boys. Girls had a higher vitamin D–sufficient status than boys (77.7% vs. 75.9%, *p* = 0.02) (Table 3). However, a significant difference between genders was not observed after adjustment for other variables (Table 1 and Table 2).

## 4. Discussion

Our results showed that 6.43% and 16.85% of children had a deficient and insufficient vitamin D status, respectively, and these problems were common in infants and children aged up to 17 years. Serum 25(OH)D was significantly associated with age, season, and visiting type. 

Children’s metabolism is fast and needs extra energy to grow. A general diet may not meet the daily vitamin D requirement [4]. Vitamin D status has a profound effect on children’s growth and development. So we mainly focused on children’s vitamin D nutrient status . Both forms of vitamin D_2_ (ergocalciferol) and vitamin D_3_ (cholecalciferol) are finally transferred into the biologically active form—1,25-dihydroxyvitamin D (1,25(OH)_2_D). The form 1,25(OH) _2_D is the most potent vitamin D metabolite. Because circulating serum 1,25(OH)_2_D is tightly regulated, concentrations of the 25(OH)D are the best measure of vitamin D nutritional status [20], and this is the major circulating form of vitamin D. 

The 25(OH)D concentration varies in different latitudes according to the duration of sunlight exposure. According to a study in Beijing, the prevalence of subclinical vitamin D deficiency (<5 ng/mL) was 45.2% in winter [16]. In Shanxi, vitamin D deficiency (<12.5 ng/mL) in the spring was found in 65.3% among children [21]. However, the prevalence of vitamin D deficiency is low in southern China. A survey in Hangzhou revealed that serum 25(OH)D concentrations less than 30 ng/mL and 20 ng/mL were seen in 33.6% and 5.4%, respectively, of infants as well as in 89.6% and 46.4%, respectively, of adolescents [22]. In Wuxi, 16.1% of the children aged one to three years were found to have a deficient vitamin D status (<20 ng/mL) [4]. In our study, 6.43% and 16.85% of the subjects had a deficient (<20 ng/mL) and insufficient (20–30 ng/mL) status. Overall, the prevalence of vitamin D deficiency and insufficiency in children was lower in Huzhou than those in other areas of China, but the present study included children aged under one year, who were supplemented with vitamin D from milk and infant formulas; it partly resulted in the low prevalence of deficient and insufficient vitamin D status. At the same time, Huzhou is located on a plain with abundant sunlight and its latitude is lower than many northern countries, which may contribute to the vitamin D status. Furthermore, the selected cut-off points to define deficiency and sufficiency vary with different prevalence [23]. The cut-offs in the present manuscript based on the Endocrine Society clinical practice guidelines were widely used in many studies. A review showed that the most advantageous serum concentrations of 25(OH)D begin at 30 ng/mL [24], which was consistent with our cut-off points.

The results of studies about serum 25(OH)D concentrations in boys and girls are inconsistent. As with most previous studies in children [8,10], we did not reveal any significant difference between gender. This could be explained by the fact that young children are more likely to play outside and have less academic pressure, as well as similar clothing practices and living habits when they start school. The prevalence of vitamin D inadequacy varies not only across countries but also with age groups. Our findings were consistent with the findings from a study among a 0–16 years age group by Andiran et al. [1], which showed that a lower vitamin D deficiency rate was found in the younger age group in Turkey. Our study was also consistent with the finding from a study in southern Iran of children nine to 18 years old, which found that children’s age was inversely related to serum 25(OH)D concentrations [25]. A study among mother-infant dyads showed that the breastfeeding infant median 25(OH)D was 8 ng/mL [26]. Another global exploration showed that added formula feeding predicted a higher infant vitamin D status [27]. Our results also showed that infants had a lower vitamin D status, while the concentration maintained a high level when children were six months to one year old, which might be due the fact that, in addition to breastfeeding, children began to feed on formula milk and complementary food after six months. However, when children grew to one year, the concentrations of vitamin D began to decline. It might because they stop the daily supplements of vitamin D from formula milk. According to the findings, we recommend that children should be reinforced with outdoor activities after the age of one year. Although the Chinese Medical Association also recommends that all children must receive 400 IU/day of vitamin D until the age of two years [28], children older than two years also need to supplement vitamin D. Additionally, when children grew to six years, we still found there was a large proportion of children with vitamin D deficiency. It might result from low exposure to sunshine during the school year. The group aged 11–17 years had the highest prevalence of low vitamin D, a marked difference between young children and adolescents could be explained by the difference in feeding, behavioral habits and lifestyle.

The sampling seasons were also associated with children’s vitamin D status. Our study revealed that the prevalence of vitamin D deficiency and insufficiency was highest in winter. Previous studies have also reported that the serum 25(OH)D concentration is lower in winter than in summer [29,30], which might because people are lack of enough sunlight expose and spent less time on outdoor exercise in a cold weather, and vitamin D supplementation is not available [31]. 

In the present study, the serum 25(OH)D concentration in clinical visiting children was significantly lower than that in health examination children (*p* < 0.05). Similar to the study in Nanjing, vitamin D deficiency occurred in 1.3% of healthy children and in 16.7% of sick children aged one to 10 years [14]. This phenomenon may be ascribed to vitamin D reinforcing immunomodulation [32] through its complicated effects on macrophages and lymphocytes [33], as 25(OH)D is a negative acute-phase reactant of disease. Children with diseases, such as infection, are more likely to have low concentrations [32]. Meanwhile, sick children have relatively reduced intake of food and less outdoor activity. This phenomenon suggests that vitamin D supplements may be necessary for children presenting when suffering from diseases. 

There were several strengths in our study. Firstly, this study represents a large sample size that covered health examination children and clinical visiting children. Secondly, the present study first evaluated vitamin D status in the whole age range of the pediatric population. However, there are still some limitations. One of the limitations in this study was the lack of children’s sociodemographic characteristics and lifestyle factors, such as dietary habits and outdoor activity time, which have effects on the serum 25(OH)D concentrations. Another limitation was that the diagnostic criteria we used to assess vitamin D status was the Endocrine Society clinical practice guidelines, and different results may be derived if different guidelines are adopted. Furthermore, children’s regular health check-ups are required by the Chinese childrens’ health policy. Though this was a hospital-based survey, the study included 8499 children who went to the hospital for routine health examinations. Therefore, the participants of the health examination were representative of the children in south China.

## 5. Conclusions

This study found that there is a high prevalence of vitamin D deficiency and insufficiency status in newborns and children aged one to 17 years in the south of China. The condition was more severe in winter, in sick children and in older children. It indicates that more sunshine and vitamin D–fortified foods are necessary among Chinese children.

## Figures and Tables

**Table 1:** The association of variables with 25(OH)D concentration (continuous value) * (*N* = 13997).

Variables	*N* (%)	Serum 25(OH)D		
		Mean ± SD	β (se)	*p* Value
Gender				
Boys	7739 (55.3)	39 ± 12	REF	
Girls	6258 (44.7)	40 ± 12	0.13(0.19)	0.50
Age				
0–3 months	1288 (9.2)	29 ± 11	REF	
4–6 months	2162 (15.5)	43 ± 11	13.27(0.42)	<0.01
7–12 months	1577 (11.3)	45 ± 11	15.29(0.44)	<0.01
1–3 years	7703 (55.0)	41 ± 11	11.27(0.39)	<0.01
4–6 years	786 (5.6)	27 ± 8	−3.25(0.45)	<0.01
7–10 years	395 (2.8)	23 ± 7	−6.79(0.50)	<0.01
11–17 years	86 (0.6)	21 ± 8	−8.97(0.87)	<0.01
Season				
Spring	3919 (28.0)	39 ± 12	REF	
Summer	4914 (35.1)	40 ± 12.	1.62(0.23)	<0.01
Autumn	2451 (17.5)	40 ± 12	1.13(0.27)	<0.01
Winter	2713 (19.4)	36 ± 12	−2.56(0.27)	<0.01
Visiting Type				
Health Examination	8499 (60.7)	41 ± 11	REF	
Clinical Visiting	5498 (39.3)	37 ± 13	−0.45(0.21)	0.03

* The generalized estimated equation model includes all variables in this table.

**Table 2:** The association * of variables with 25(OH)D concentration (categorical variable) (*N* = 13997).

Variables	Sufficiency	Insufficiency			Deficiency		
	*N* (%)	N (%)	OR (95%CI)	*p* Value	*N* (%)	OR(95%CI)	*p* Value
**Gender**							
Boys	5874 (54.7)	1364 (57.9)	REF		501 (55.7)	REF	
Girls	4865 (45.3)	994 (42.2)	0.96 (0.87-1.05)	0.35	399 (44.3)	1.14 (0.98-1.33)	0.10
**Age**							
0–3 months	570 (5.3)	424 (18.0)	REF		294 (32.7)	REF	
4–6 months	1926 (17.9)	179 (7.6)	0.14 (0.11–0.17)	<0.01	57 (6.3)	0.06 (0.05–0.09)	<0.01
7–12 months	1436 (13.4)	114 (4.8)	0.12 (0.10–0.15)	<0.01	27 (3.0)	0.04 (0.03–0.06)	<0.01
1–3 years	6466 (60.2)	1064 (45.1)	0.26 (0.22–0.31)	<0.01	173 (19.2)	0.06 (0.05–0.08)	<0.01
4–6 years	264 (2.5)	352 (14.9)	2.14 (1.74–2.64)	<0.01	170 (18.9)	1.66 (1.29–2.14)	<0.01
7–10 years	70 (0.6)	187 (7.9)	4.32 (3.18–5.87)	<0.01	138 (15.3)	5.31 (3.81–7.41)	<0.01
11–17 years	7 (0.1)	38 (1.6)	8.57 (3.78–19.44)	<0.01	41 (4.6)	14.61 (6.41–33.27)	<0.01
**Season**							
Spring	3004 (28.0)	638 (27.0)	REF		277 (30.8)	REF	
Summer	3864 (36.0)	796 (33.8)	0.78 (0.69–0.89)	<0.01	254 (28.2)	0.49 (0.41–0.60)	<0.01
Autumn	1964 (18.3)	371 (15.7)	0.80 (0.69–0.93)	<0.01	116 (12.9)	0.53 (0.42–0.68)	<0.01
Winter	1907 (17.7)	553 (23.5)	1.37 (1.20–1.58)	<0.01	253 (28.1)	1.33 (1.09–1.63)	0.01
**Visiting Type**							
Health Examination	7005 (65.2)	1170 (49.6)	REF		324 (36.0)	REF	
Clinical Visiting	3734 (34.8)	1188 (50.4)	1.19 (1.07–1.33)	<0.01	576 (64.0)	1.23 (1.03–1.47)	0.03

* The multiple logistic regression model includes all variables in this table.

**Table 3:** The distribution of relevant variables with different levels of serum 25(OH)D (*N* = 13997).

Variables	Distribution of Serum 25(OH)D			*p* Value
	Sufficiency (*n* = 10,739)	Insufficiency (*n* = 2358)	Deficiency (*n* = 900)	
*N* (%)				
**Gender** ^a^				
Boys	5874 (75.9)	1364 (17.6)	501 (6.5)	0.02
Girls	4865 (77.7)	994 (15.9)	399 (6.4)	
**Season** ^a^				
Spring	3004 (76.6)	638 (16.3)	277 (7.1)	<0.01
Summer	3864 (78.6)	796 (16.2)	254 (5.2)	
Autumn	1964 (80.2)	371 (15.1)	116 (4.7)	
Winter	1907 (70.3)	553 (20.4)	253 (9.3)	
**Visiting Type** ^a^				
Health Examination	7005 (82.4)	1170 (13.8)	324 (3.8)	<0.01
Clinical Visiting	3734 (67.9)	1188 (21.6)	576 (10.5)	
Mean ± SD				
**Age, years** ^b^	3 ± 4	3 ± 3	1 ± 1	<0.01
**25(OH)D, ng/mL** ^b^	44 ± 9	26 ± 3	1 ± 4	<0.01

^a^ Values were compared using Chi Square(χ^2^) test for the categorical variables; ^b^ Values were compared using analysis of variance (ANOVA) for the continuous variables.

## Supplementary Materials

The following are available online at http://www.mdpi.com/2072-6643/9/4/319/s1, Table S1: The association of variables and vitamin D (continuous variable) stratified by visiting type, Table S2: The association of variables and vitamin D (categorical variable) in clinical visiting children (*N* = 5498), Table S3: The association of variables and vitamin D (categorical variable) in health examination children (*N* = 8499).
