# Supplementary material for: Nutrient Status of Vitamin D among Chinese Children
Source: Nutrients. 2017 Mar 23;9(4):319. doi: 10.3390/nu9040319 (PMC5409658; doi:10.3390/nu9040319)
Supplement: Supplementary file 1 [file nutrients-09-00319-s001.doc]

| **Supplementary Table S1.** The association of variables and vitamin D (continuous variable) stratified by visiting type*. | | | | | | | | |
| --- | --- | --- | --- | --- | --- | --- | --- | --- |
| **Variables** | **Clinical visiting children (*N* = 5498)** | | | | **Health examination children (*N* = 8499)** | | | |
|  | ***N* (%)** | **Serum 25(OH)D** | | | ***N* (%)** | **Serum 25(OH)D** | | |
|  |  | **Mean ± SD** | **β(se)** | ***P* Value** |  | **Mean ± SD** | **β(se)** | ***P* Value** |
| **Gender** |  |  |  |  |  |  |  |  |
| Boys | 3147(57.2) | 36 ± 13 | REF |  | 4592(54.0) | 41 ± 11 | REF |  |
| Girls | 2351(42.8) | 37 ± 13 | 0.38(0.31) | 0.22 | 3907(46.0) | 41 ± 12 | −0.02(0.24) | 0.93 |
| **Age** |  |  |  |  |  |  |  |  |
| 0–3 months | 1202(21.9) | 29 ± 11 | REF |  | 86(1.0) | 32 ± 12 | REF |  |
| 4–6 months | 741(13.5) | 42 ± 11 | 13.01(0.54) | <0.01 | 1421(16.7) | 43 ± 10 | 11.28(1.27) | <0.01 |
| 7–12 months | 654(11.9) | 45 ± 11 | 15.68(0.55) | <0.01 | 923(10.9) | 45 ± 10 | 12.89(1.29) | <0.01 |
| 1–3 years | 2163(39.3) | 41 ± 11 | 11.67(0.43) | <0.01 | 5540(65.2) | 41 ± 11 | 8.93(1.25) | <0.01 |
| 4–6 years | 433(7.9) | 27 ± 8 | −2.75(0.51) | <0.01 | 353(4.1) | 27 ± 8 | −5.79(1.32) | <0.01 |
| 7–10 years | 257(4.6) | 24 ± 7 | −5.91(0.58) | <0.01 | 138(1.6) | 22 ± 7 | −10.15(1.37) | <0.01 |
| 11–17 years | 48(0.9) | 22 ± 7 | −7.19(1.08) | <0.01 | 38(0.5) | 19 ± 8 | −13.25(1.72) | <0.01 |
| **Season** |  |  |  |  |  |  |  |  |
| Spring | 1593(29.0) | 37 ± 13 | REF |  | 2326(27.4) | 41 ± 12 | REF |  |
| Summer | 1972(35.9) | 38 ± 13 | 2.19(0.37) | <0.01 | 2942(34.6) | 42 ± 11 | 1.23(0.30) | <0.01 |
| Autumn | 858(15.6) | 38 ± 13 | 2.04(0.46) | <0.01 | 1593(18.7) | 41 ± 11 | 0.54(0.34) | 0.12 |
| Winter | 1075(19.5) | 34 ± 13 | −1.19(0.44) | 0.01 | 1638(19.3) | 38 ± 11 | −3.47(0.34) | <0.01 |
| *The generalized estimated equation model includes all variables in this table. | | | | | | | | |

| **Supplementary Table S2.** The association of variables and vitamin D (categorical variable) in clinical visiting children* (*N* = 5498). | | | | | | | |
| --- | --- | --- | --- | --- | --- | --- | --- |
| **Variables** | **Sufficiency** | **Insufficiency** | | | **Deficiency** | | |
|  | ***N* (%)** | ***N* (%)** | **OR(95%CI)** | ***P* Value** | ***N* (%)** | **OR(95%CI)** | ***P* Value** |
| **Gender** |  |  |  |  |  |  |  |
| Boys | 2085(55.8) | 718(60.4) | REF |  | 344(59.7) | REF |  |
| Girls | 1649(44.2) | 470(39.6) | 0.908(0.78–1.05) | 0.20 | 232(40.3) | 0.98(0.80–1.19) | 0.80 |
| **Age** |  |  |  |  |  |  |  |
| 0–3 months | 524(14.0) | 396(33.3) | REF |  | 282(49.0) | REF |  |
| 4–6 months | 623(16.7) | 84(7.1) | 0.18(0.14–0.23) | <0.01 | 34(5.9) | 0.10(0.07–0.15) | <0.01 |
| 7–12 months | 592(15.8) | 50(4.2) | 0.11(0.08–0.16) | <0.01 | 12(2.1) | 0.04(0.02–0.07) | <0.01 |
| 1–3 years | 1799(48.2) | 303(25.5) | 0.23(0.19–0.28) | <0.01 | 61(10.6) | 0.07(0.05–0.09) | <0.01 |
| 4–6 years | 142(3.8) | 205(17.3) | 2.05(1.59–2.64) | <0.01 | 86(14.9) | 1.32(0.97–1.80) | 0.08 |
| 7–10 years | 48(1.3) | 126(10.6) | 3.75(2.61–5.38) | <0.01 | 83(14.4) | 3.89(2.63–5.76) | <0.01 |
| 11–17 years | 6(0.2) | 24(2.0) | 5.52(2.23–13.65) | 0.02 | 18(3.1) | 6.09(2.37–15.60) | <0.01 |
| **Season** |  |  |  |  |  |  |  |
| Spring | 1073(28.7) | 339(28.5) | REF |  | 181(31.4) | REF |  |
| Summer | 1382(37.0) | 422(35.5) | 0.76(0.63–0.91) | <0.01 | 168(29.2) | 0.53(0.41–0.68) | <0.01 |
| Autumn | 623(16.7) | 171(14.4) | 0.73(0.58–0.92) | 0.01 | 64(11.1) | 0.49(0.35–0.68) | <0.01 |
| Winter | 656(17.6) | 256(21.6) | 1.05(0.85–1.29) | 0.66 | 163(28.3) | 1.07(0.83–1.40) | 0.59 |
| *The multiple logistic regression model includes all variables in this table. | | | | | | | |

| **Supplementary Table S3.** The association of variables and vitamin D (categorical variable) in health examination children* (*N* = 8499). | | | | | | | |
| --- | --- | --- | --- | --- | --- | --- | --- |
| **Variables** | **Sufficiency** | **Insufficiency** | | | **Deficiency** | | |
|  | ***N* (%)** | ***N* (%)** | **OR(95%CI)** | ***P* Value** | ***N* (%)** | **OR(95%CI)** | ***P* Value** |
| **Gender** |  |  |  |  |  |  |  |
| Boys | 3789(54.1) | 646(55.2) | REF |  | 157(48.5) | REF |  |
| Girls | 3216(45.9) | 524(44.8) | 0.99(0.87–1.13) | 0.85 | 167(51.5) | 1.43(1.11–1.83) | <0.01 |
| **Age** |  |  |  |  |  |  |  |
| 0–3 months | 46(0.7) | 28(2.4) | REF |  | 12(3.7) | REF |  |
| 4–6 months | 1303(18.6) | 95(8.1) | 0.11(0.07–0.19) | <0.01 | 23(7.1) | 0.06(70.03–0.13) | <0.01 |
| 7–12 months | 844(12.1) | 64(5.5) | 0.13(0.08–0.22) | <0.01 | 15(4.6) | 0.07(0.03–0.17) | <0.01 |
| 1–3 years | 4667(66.6) | 761(65.0) | 0.28(0.17–0.45) | <0.01 | 112(34.6) | 0.10(0.05–0.19) | <0.01 |
| 4–6 years | 122(1.7) | 147(12.6) | 2.23(1.31–3.81) | <0.01 | 84(25.9) | 3.48(1.71–7.08) | <0.01 |
| 7–10 years | 22(0.3) | 61(5.2) | 5.36(2.71–10.61) | <0.01 | 55(17.0) | 13.76(6.03–31.41) | <0.01 |
| 11–17 years | 1(0.0) | 14(1.2) | 26.81(3.33–215.55) | <0.01 | 23(7.1) | 126.86(15.31–>999.99) | <0.01 |
| **Season** |  |  |  |  |  |  |  |
| Spring | 1931(27.6) | 299(25.5) | REF |  | 96(29.6) | REF |  |
| Summer | 2482(35.4) | 374(32.0) | 0.80(0.68–0.95) | 0.01 | 86(26.6) | 0.42(0.30–0.59) | <0.01 |
| Autumn | 1341(19.1) | 200(17.1) | 0.86(0.70–1.05) | 0.14 | 52(16.0) | 0.57(0.39–0.84) | <0.01 |
| Winter | 1251(17.9) | 297(25.4) | 1.68(1.40–2.02) | <0.01 | 90(27.8) | 1.71(1.23–2.37) | <0.01 |
| *The multiple logistic regression model includes all variables in this table. | | | | | | | |
